# Chronic "*Candidatus *Mycoplasma turicensis" infection

**DOI:** 10.1186/1297-9716-42-59

**Published:** 2011-04-20

**Authors:** Marilisa Novacco, Felicitas S Boretti, Godelind A Wolf-Jäckel, Barbara Riond, Marina L Meli, Barbara Willi, Hans Lutz, Regina Hofmann-Lehmann

**Affiliations:** 1 Clinical Laboratory, Vetsuisse Faculty, University of Zurich, Winterthurerstrasse 260, 8057 Zurich, Switzerland; 2 Clinical for Small Animal Internal Medicine, University of Zurich, Winterthurerstrasse 260, 8057 Zurich, Switzerland

## Abstract

"*Candidatus *Mycoplasma turicensis" infects felids. The pathogenesis of "*Candidatus *M. turicensis" chronic infection is poorly understood. The goals of the present study were to (1) induce reactivation of the infection in chronic carrier cats by attempted immunosuppression, (2) identify potential tissue sequestration using real-time TaqMan^® ^PCR and (3) monitor the humoral immune response by DnaK enzyme-linked immunosorbent assay (ELISA). Ten specified pathogen-free cats that had ostensibly recovered from experimental "*Candidatus *M. turicensis" infection were used: five cats (group 1) received high dose methylprednisolone (attempted immunosuppression), while five cats served as untreated controls (group 2). Besides weekly blood samples, tissue samples were collected from bone marrow, kidney, liver and salivary glands at selected time points. The cats in group 1 had significantly lower lymphocyte counts and higher blood glucose levels after methylprednisolone administration than the controls. After methylprednisolone administration one blood and three tissue samples from cats in group 1 tested PCR-positive; before the administration, only one sample was positive. All other samples tested PCR-negative. All cats stayed seropositive; the antibody levels of the cats in group 1 showed a significant transient decrease after methylprednisolone administration. This is the first study to report the presence of "*Candidatus *M. turicensis" in tissues of chronically infected cats and the persistence of anti-feline hemoplasma antibodies in the absence of detectable bacteremia. Methylprednisolone administration did not lead to a significant reactivation of the infection. Our results enhance the knowledge of "*Candidatus *M. turicensis" infection pathogenesis and are clinically relevant to the prognosis of hemoplasma-infected cats.

## Introduction

Hemotropic mycoplasmas, also known as hemoplasmas, are small, uncultivable, cell-wall-free bacteria that attach to red blood cells. Hemoplasmas are the causative agents of infectious anemia in many mammalian species. In domestic cats, three hemoplasma species have been identified, which differ in their pathogenic potential [1]: *Mycoplasma haemofelis *(*M. haemofelis*), "*Candidatus *M. haemominutum" and "*Candidatus *Mycoplasma turicensis" ("*Candidatus *M. turicensis"). "*Candidatus *M. turicensis" was identified in a Swiss cat with hemolytic anemia [2]. During the acute phase of the infection, "*Candidatus *M. turicensis" can induce mild to moderate anemia in experimentally infected domestic cats [2-4]. After bacteremia, hemoplasma-infected cats may become chronic carriers [5]. To date, it is assumed that infected animals do not completely clear the "*Candidatus *M. turicensis" organisms, even after antibiotic treatment [6-8]. Different studies have suggested a possible sequestration of feline hemoplasmas in tissues [9-11]. The dynamics of the chronic phase of hemoplasma infection, however, are still poorly understood. We hypothesized that chronically infected cats may be able to reactivate the infection under particular conditions, such as immunosuppression. Chronic carrier cats could subsequently represent a source of infection for other animals. To date, no data from long-term follow-up studies of experimental feline hemoplasma infection have been reported.

Thus, the goals of the present study were to (1) induce and investigate the reactivation of chronic experimental "*Candidatus *M. turicensis" infection, (2) identify potential sequestration sites prior to and during the attempted reactivation of the infection in chronic carrier cats and (3) monitor the humoral immune response throughout the experiment.

## Materials and methods

### Animals and experimental design

The present study was conducted with ten specified pathogen-free (SPF) male castrated cats. They had undergone acute experimental "*Candidatus *M. turicensis" infection after subcutaneous inoculation of "*Candidatus *M. turicensis"-positive blood in a previous experiment [3]. The cats were kept in groups in a confined university facility under ideal ethological conditions as described [12]. All of the experiments were performed according to the law and were officially approved by the veterinary office of the canton Zurich (TVB 101/2007). At three months after the experimental "*Candidatus *M. turicensis" infection, all cats tested "*Candidatus *M. turicensis"-negative in the blood [3] as determined by polymerase chain reaction (PCR). The cats were assigned to the present study 12 to 17 months after the experimental infection. For this purpose, the ten cats were divided into two groups of five: group 1 (cats A2, R2, S1, T1 and X4) received methylprednisolone and group 2 (cats A1, R1, S2, T2 and X5) served as untreated control cats. The cats underwent regular clinical examination, and their body weights and temperatures were recorded. The cats were monitored for twenty-two consecutive weeks after methylprednisolone administration.

### Methylprednisolone administration and tissue sample collection

In an attempt to immunocompromise the cats and induce the *in vivo *reactivation of the "*Candidatus *M. turicensis" infection, the cats in group 1 received three times a high dose of methylprednisolone acetate (10 mg/kg body weight, i.m., Depo-Medrol ad us. vet., Pfizer AG, Zurich, Switzerland) in three consecutive weeks (first injection = day 0, second and third injection at days 7 and 14, respectively). Ethylenediaminetetraacetic acid (EDTA)-anticoagulated blood and serum samples were collected from all ten cats prior to methylprednisolone administration and weekly thereafter for hematology, PCR analysis, serology and, where clinically indicated, serum biochemistry. The samples for PCR and serology were stored at -80°C within two hours of collection. Complete hemograms were performed using a Sysmex XT-2000iV (Sysmex Corporation, Kobe, Japan) [13]. Packed cell volume (PCV) values between 33-45% were considered to be within the reference range, and anemia was defined as a PCV <33%. For the white blood cell differential, microscopic blood smear evaluation was performed. Two blood smears were stained with a modified Wright-stain on an automated staining instrument for each blood sample (Hema Tek 1000, Bayer AG, Zurich, Switzerland). Two technicians with more than ten years of experience in veterinary hematology differentiated independently 100 cells per smear. Blood glucose concentrations were determined daily to monitor the potential development of diabetes mellitus induced by methylprednisolone. These tests were carried out using the marginal ear vein prick technique using a portable blood glucose device (Accu-Check *Aviva*, Roche Diagnostic AG, Basel, Switzerland) [14]. The measurements were stopped 45 days after the first methylprednisolone administration when the glucose values of all cats were within the reference range (4-9 mmol/L).

Samples from the kidney, liver and salivary glands were collected by fine needle aspiration (FNA) 14 days prior to methylprednisolone administration and 1 and 4 weeks after the end of the methylprednisolone administration (i.e., at days 20 and 42). In addition, saliva swabs and bone marrow aspirates were collected. Bone marrow aspirations were performed on the proximal humerus as described [3]. All fine needle and bone marrow aspirations were performed under a short-duration general anesthetic using a previously described protocol [3]. Deoxyribonucleic acid (DNA) was either extracted immediately after the tissue collection, or the samples were frozen at -80°C within two hours of the collection.

### Nucleic acid extractions

The total nucleic acid (TNA) was extracted from 100 μL EDTA-anticoagulated blood using the MagNaPure LC Total Nucleic Acid Isolation Kit (Roche Diagnostics, Rotkreuz, Switzerland). The TNA was eluted in 100 μL of elution buffer and stored at -80°C until PCR was performed. Nucleic acid was extracted from tissue samples using the DNA Micro kit (Qiagen, Hombrechtikon, Switzerland). Saliva swabs and bone marrow samples were extracted using the DNA blood and tissue kit (Qiagen) according to the manufacturer's instructions. The DNA was eluted into 100 μL buffer AE and stored at -80°C until use. During all extractions, negative controls consisting of 200 μL of phosphate-buffered saline were concurrently prepared with each batch of samples to monitor for cross-contamination.

### Quantitative TaqMan^® ^real-time PCR assays

The tissue samples were analyzed using a TaqMan^® ^real-time PCR amplifying glyceraldehyde-3-phosphate dehydrogenase (GAPDH) pseudogene [15] to test for the presence of amplifiable DNA and the absence of significant PCR inhibitors as previously described [16]. Only samples with more than 5 000 copies of GAPDH per reaction were considered to be sufficient, and the extraction was repeated for samples with insufficient copies. The "*Candidatus *M. turicensis" loads were quantified in all blood and tissue samples (in triplicate) by TaqMan^® ^real-time PCR on an ABI PRISM 7700 Sequence Detection System (Applied Biosystems, Rotkreuz, Switzerland) as previously described [2]. The "*Candidatus *M. turicensis" copy numbers per cell in the tissue were calculated by dividing the "*Candidatus *M. turicensis" copy numbers by the GAPDH copy numbers [17]. The detection limit of the "*Candidatus *M. turicensis" real-time PCR was 200 copies/mL of blood and 66 copies/10^6 ^cells.

### Serology

Antibodies to "*Candidatus *M. turicensis" were assessed in serum samples using an enzyme-linked immunosorbent assay (ELISA) and a recombinant DnaK protein as previously described [18]. We used a serum dilution of 1:100 and 50 ng of the recombinant protein per well. The signal-to-noise ratio was calculated by dividing the postinfection absorbance by the preinfection absorbance values for each individual cat [18]. An ELISA signal-to-noise ratio of ≥1.5 was considered to be positive [18].

### Statistics

Statistical analyses were performed using the Excel add-in Analyse-it (Analyse-it Software, Leeds, UK), and we used nonparametric tests. The parameters were compared between two groups using the Mann-Whitney U-test (*P*a_MWU_). The Wilcoxon signed-rank test (*P*_Wilcoxon_) was used to compare "*Candidatus *M. turicensis" antibody levels from the same cat at two different time points, and Friedman's test (*P_Friedman_*) was used to analyze the parameters over time when more than two time points were considered. Frequencies were compared using the chi-square test (*P_chi_*^2^) and Fisher's exact test (*P_Fisher_*). *P *values < 0.05 were considered to be significant. All reference ranges of clinical parameters are given as 5% and 95% quantiles, which were determined from 58 clinically healthy cats using the same methods applied in the current study.

## Results

### "*Candidatus *M. turicensis" blood and tissue loads

At the beginning of this project, the blood of all ten cats was "*Candidatus *M. turicensis"-negative as determined by real-time PCR. After methylprednisolone administration, all of the blood samples tested "*Candidatus *M. turicensis"-negative in PCR except one sample collected from cat S1 (group 1) one week after the end of methylprednisolone administration (at day 20, Table 1). The "*Candidatus *M. turicensis" blood load of the latter sample was low (800 copies/mL of blood, Table 1), which corresponded to 1 copy/10^7 ^red blood cells. The blood samples of all five cats in group 2 remained PCR-negative throughout the experiment. In the tissue samples, a sufficient amount of amplifiable DNA and the absence of significant PCR inhibition were confirmed using GAPDH real-time PCR. The majority of the tissue samples were PCR-negative for "*Candidatus *M. turicensis" (Table 1). Before methylprednisolone administration, one cat in group 1 (cat R2) was PCR-positive for "*Candidatus *M. turicensis" in the salivary gland. At day 20 (one week after the end of methylprednisolone administration), the tissue samples of two cats in group 1 were PCR-positive for "*Candidatus *M. turicensis": cat R2 in the salivary gland and cat T1 in the liver (Table 1). Cat R2, which tested PCR-positive twice, showed an approximately 40-fold higher "*Candidatus *M. turicensis" tissue load in the salivary gland at day 20 compared with the "*Candidatus *M. turicensis" load prior to methylprednisolone administration (Table 1). Overall, three out of the five cats that received methylprednisolone showed an increase in "*Candidatus *M. turicensis" tissue or blood loads (*P*_chi_^2 ^= 0.0384; *P*_Fisher _= 0.1667). By day 42 (four weeks after the end of the methylprednisolone administration), however, all of the cats in group 1 were PCR-negative in all the tissues analyzed. None of the tissue samples from the control cats in group 2 tested "*Candidatus *M. turicensis"-positive by PCR.

**Table 1:** CMt TaqMan^® ^real-time PCR results of blood and tissue samples collected prior to and after the methylprednisolone administrations

Group	Cat	Before MPA^a ^ (day -14)	1 week after end MPA (day 20)	4 weeks after end MPA (day 42)
1	A2	-^b^	-	-
	R2	+^c ^salivary gland (264^d^)	+ salivary gland (10 250 ^d^) + liver (236 ^d^)	-
	S1	-	+ blood (800^e^)	-
	T1	-	+ liver (838 ^d^)	-
	X4	-	-	-
2	A1	-	-	-
	R1	-	-	-
	S2	-	-	-
	T2	-	-	-
	X5	-	-	-

^a ^MPA = methylprednisolone administrations; ^b ^- = all samples tested PCR-negative; ^c ^+ = at least one PCR-positive result; the positive tissues are listed; ^d ^tissue loads: copies/10^6 ^cells; ^e ^blood loads: copies/mL of blood.

### Clinical and laboratory parameters

**Figure 1:**
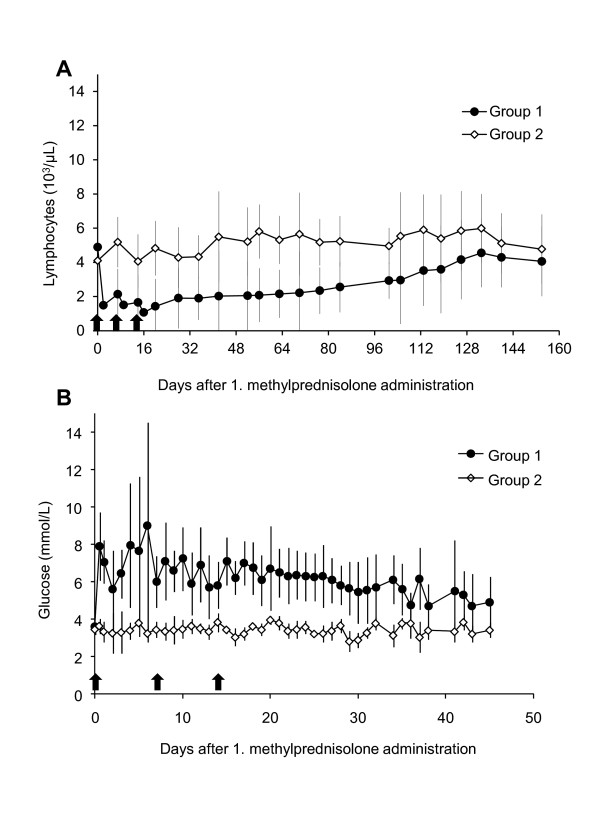
**Lymphocyte counts (A) and blood glucose levels (B) of cats in groups 1 and 2**. Values are depicted as mean +/- standard deviation. Black arrows = methylprednisolone administration. Cats in group 1 received the methylprednisolone administrations; cats in group 2 served as untreated control animals. A) Cats in group 1 had significantly lower lymphocyte counts than cats in group 2 (p_MWU _< 0.05) starting after the first methylprednisolone administration until day 70. B) Cats in group 1 had significantly higher blood glucose levels than cats in group 2 (p_MWU _< 0.05) starting after the first methylprednisolone administration at all collection time points with the exceptions of days 2, 11, and 36.

None of the cats developed anemia throughout the experiment. Cats in group 1 had lymphocyte counts below the reference range (1 050-6 000 cells/μL) at different time points after they received methylprednisolone: two cats (S1 and X4) at day 9, one cat (X4) at day 14, three cats (A2, R2, T1) at day 16 and one cat (A2) at day 20. Moreover, the cats in group 1 had significantly lower lymphocyte counts after the first methylprednisolone administration compared with the untreated control cats in group 2 (*P_MWU _*< 0.05; Figure 1A); the counts remained significantly lower until day 70 (Figure 1A). In addition, the cats in group 1 had significantly higher blood glucose levels compared with the cats in group 2 (*P*_MWU _< 0.05). This difference began after the first methylprednisolone administration and was observed at almost all collection time points (the exceptions were days 2, 11, and 36) (Figure 1B). There were no significant differences between the two groups of cats in erythrocyte counts, total leukocyte counts, hematocrit, body temperature or weight.

### Humoral immune response

**Figure 2:**
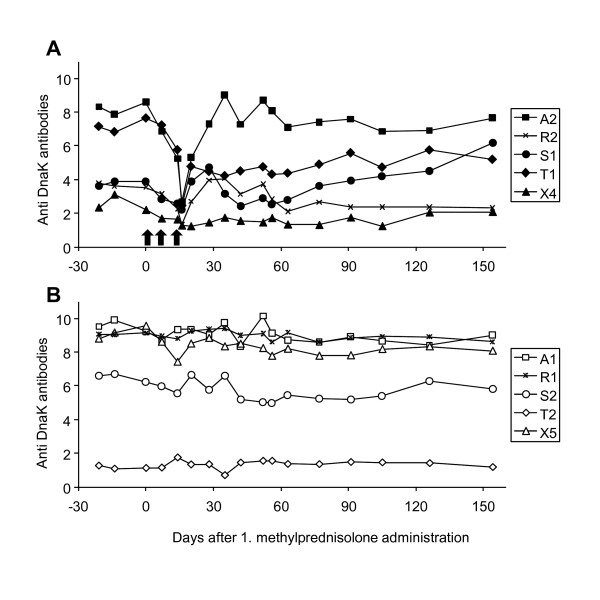
**Antibody levels to DnaK depicted as ELISA signal-to-noise ratio**. A) Cats in group 1; methylprednisolone administration (black arrows); B) cats in the untreated control group 2.

All cats were seropositive at the start of the study (ELISA signal-to-noise ratio ≥1.5). Six cats had high antibody levels (ELISA signal-to-noise ratio >6), three cats had intermediate levels (ELISA signal-to-noise ratio between 2 and 4) and one cat had low antibody levels (ELISA signal-to-noise ratio <2) (Figure 2). There was no significant difference between the antibody levels of groups 1 and 2 at the beginning of the present study. A significant decrease in antibody levels (*P_Friedman_*= 0.0018) was observed throughout methylprednisolone administration (from day 0 to 16) in group 1. This decrease was followed by a significant increase (from day 16 to 35, *P*_Friedman _= 0.0452), which returned the antibody levels to approximately pretreatment levels (Figure 2A). In the untreated control group, no significant changes in the antibody levels were found during these periods (Figure 2B). Moreover, no significant differences in the antibody levels were observed between groups 1 and 2 at any of the time points tested (17 blood collections throughout the observation period); however, a trend towards difference was found at day 20 when the cats in group 1 had slightly lower antibody levels than the cats in group 2 (*P*_MWU _= 0.0952). In addition, when the antibody levels from all ten cats were compared between day 0 and the end of the project (day 154), we found a trend toward a decrease (*P*_Wilcoxon _= 0.0840). In contrast, in cat S1, which was the only cat that was transiently blood PCR-positive, we found an increase in the antibody levels from day 35 until the end of the experiment, and the antibody level was higher at the end of the experiment than it was at the beginning.

## Discussion

The present study was the first to investigate the chronic phase of experimental "*Candidatus *M. turicensis" infection. We documented the detection of "*Candidatus *M. turicensis" in tissues in the absence of detectable "*Candidatus *M. turicensis" bacteremia and demonstrated the persistence of antibodies to "*Candidatus *M. turicensis" many months after exposure. In addition, attempted immunosuppression did not lead to a significant reactivation of the infection under the conditions chosen.

Recently, Tasker et al. analyzed blood and tissue copy number distribution during experimental *M. haemofelis *infection [11]. Tissue sequestration was mainly investigated to explain the marked variation in blood copy numbers (4 log difference over 2 or 3 days) that can be observed during *M. haemofelis *infection. The Tasker et al. study failed to demonstrate significant tissue sequestration of *M. haemofelis *at the time points investigated [11].

Studies have shown that the "*Candidatus *M. turicensis" loads do not fluctuate very much in blood and "*Candidatus *M. turicensis" blood loads have been shown to decrease below the PCR detection level earlier than *M. haemofelis *blood loads [2,3]. At the beginning of the present study (12 to 17 months post-"*Candidatus *M. turicensis" inoculation), the blood from all ten cats tested PCR-negative for "*Candidatus *M. turicensis"; thus, no significant contribution of the "*Candidatus *M. turicensis" blood loads to tissue loads was expected. In the present study the tissue samples were collected using fine needle aspiration (FNA); it has been demonstrated that material collected by FNA is appropriate for the detection of various other infectious pathogens by PCR [19-22] and PCR from specimens collected by FNA may be more sensitive than PCR from blood [21] or other detection methods [22]. The presence of sufficient material (amplifiable DNA) was confirmed for all FNA samples by feline GAPDH PCR. The detection of "*Candidatus *M. turicensis" in the liver and salivary gland indicated that "*Candidatus *M. turicensis" was still present in these cats more than one year after exposure. Currently, there is no *in vitro *test system to assess the viability of hemoplasmas. PCR-positive results do not provide information regarding the presence of viable organisms; however, it is generally believed that the DNA of dead organisms is immediately cleared from the body [9]. Therefore, the ~40-fold increase in "*Candidatus *M. turicensis" copies in the salivary gland of cat R2, combined with the PCR-positive results of a tissue sample and a blood sample from two additional cats (cat S1 and T1) after methylprednisolone administration, supports the hypothesis that there were still viable organisms in these cats.

The "*Candidatus *M. turicensis" tissue loads appeared to be indirectly associated with the elapsed time since infection. The cats examined in the present study also underwent a preliminary tissue collection 6 to 11 months after "*Candidatus *M. turicensis" infection. At that time, "*Candidatus *M. turicensis" was present in three (cats R2, T1 and X4) of the ten cats (one kidney, two liver and two bone marrow samples tested positive; data not shown). At the beginning of the present study and prior to methylprednisolone administration (12 to 17 months after the "*Candidatus *M. turicensis" infection), only one tissue of one cat was PCR-positive, and, at the end of this project (18 to 23 months after infection), all the animals showed negative PCR results in all tissue samples.

Currently, PCR analysis is the method of choice to diagnose hemoplasma infections. During the chronic phase of the infection, however, the hemoplasmas are present at very low levels in the blood and tissues. Thus, the detection of hemoplasmas in chronically infected cats is problematic because the bacterial loads are at, or below, the detection limit of real-time PCR. Therefore, a PCR-negative result cannot rule out a low-level infection. The recently developed ELISAs [18,23] offer the possibility to monitor and quantify the humoral immune response during chronic "*Candidatus *M. turicensis" infection. Because all of the cats in the present study showed persistently high antibody levels throughout the entire experiment, complete tissue clearance of the organisms seemed unlikely. The constant presence of the antigen may continuously stimulate the immune system, which in turn keeps the bacterial copy numbers at very low levels. Indeed, after methylprednisolone administration, we found a significant decrease in the antibody levels, which was associated with an increase in the number of detectable "*Candidatus *M. turicensis" copy numbers as determined by PCR.

The reactivation of feline hemoplasma infection by corticosteroid administration has been attempted in *M. haemofelis*-infected cats treated with antibiotics [6-8], but these studies did not have a long period between infection and the attempted reactivation. Although antibiotics were effective at reducing *M. haemofelis *blood loads, they did not completely eliminate the bacteria and a reactivation of *M. haemofelis *infection could be provoked [6-8]. In addition, Harvey and colleagues tried to induce relapses of hemoplasmosis in *M. haemofelis*-infected cats using splenectomy and glucocorticoid administration [24]. The authors observed moderate and irregular bacteremia, which was determined by cytological examination, but they did not observe any clinical signs [24]. The present study was the first to report the attempted reactivation of "*Candidatus *M. turicensis" infection. We chose a methylprednisolone dose that has been successfully used to reactivate feline leukemia virus (FeLV) infection in cats and was reported to inhibit lymphocyte blastogenesis in adult cats [25]. Indeed, the cats that received methylprednisolone in the present study demonstrated significantly lower lymphocyte counts in the peripheral blood compared with the control cats. Thus, we assumed that the dose of methylprednisolone used in the present study was sufficient to induce transient immune suppression in the cats. We were unable to demonstrate significant reactivation of the "*Candidatus *M. turicensis" infection in the present study, which may have been due to the duration of the infection. Reactivation might have been more successful at an earlier time point when more cats were still tissue PCR-positive. Alternatively, differences in infection kinetics between *M. haemofelis *and "*Candidatus *M. turicensis" may play a role and immunosuppression may not lead to the reactivation of "*Candidatus *M. turicensis" infection.

Corticosteroid therapy has been reported to affect humoral immunity. In particular, corticosteroids may reversibly decrease B cell counts and specific antibody responses [26,27]. Similarly, the cats in group 1 of the present study showed a significant decrease in their antibody levels immediately after methylprednisolone administration, however, the reduction was transient. To the best of our knowledge, this was the first study to show under well-controlled conditions the effect of methylprednisolone on the antibody levels of cats.

In conclusion, the results of the present study contribute to the understanding of the pathogenesis of "*Candidatus *M. turicensis" infection. Although chronically infected cats recovered from bacteremia, we demonstrated that "*Candidatus *M. turicensis" remained detectable by PCR in tissues at low levels for many months. In addition, antibodies to "*Candidatus *M. turicensis" remained at high levels in at least some of the cats throughout the entire monitoring period. The attempted immunosuppression did not lead to a significant recurrence of bacteremia and disease at this stage of infection. Thus, chronically "*Candidatus *M. turicensis" infected cats do not appear to be a major source of infection for other cats.

## Competing interests

The authors declare that they have no competing interests.

## Authors' contributions

MN participated in the design of the study, performed the *in vivo *experiments, carried out the molecular and serological studies and drafted the manuscript. FB participated in the design of the study and the *in vivo *experiments. GWJ helped with the *in vivo *experiment and developed the immunoassay. BR was responsible for the SPF cats, housing, and the blood collections and revised the manuscript. MM supported the molecular biology work and revised the manuscript. BW revised the manuscript. HL contributed to the study design. RHL conceived the study, participated in the design and coordination of the study, performed the statistical analysis and revised the manuscript. All authors read and approved the final manuscript.
